# Effects of Short-Term Golden Root Extract (*Rhodiola rosea*) Supplementation on Resistance Exercise Performance

**DOI:** 10.3390/ijerph18136953

**Published:** 2021-06-29

**Authors:** Tyler D. Williams, Haley N. Langley, Caleb C. Roberson, Rebecca R. Rogers, Christopher G. Ballmann

**Affiliations:** Department of Kinesiology, Samford University, 800 Lakeshore Dr, Birmingham, AL 35229, USA; twilli11@samford.edu (T.D.W.); hlangley@samford.edu (H.N.L.); crobers1@samford.edu (C.C.R.); rrogers1@samford.edu (R.R.R.)

**Keywords:** adaptogen, bench press, velocity, lactate, epinephrine, norepinephrine

## Abstract

The purpose of this study was to examine the effects of short-term Golden Root Extract (GRE; *Rhodiola rosea*) supplementation on blood lactate, catecholamines, and performance during repeated bench press exercise. Resistance-trained males (*n* = 10) participated in this study. In a double-blinded, crossover, counterbalanced study design, participants supplemented with either 1500 mg/day of GRE or placebo (PL; gluten-free cornstarch) for 3 days prior to experimentation. An additional 500 mg dose was ingested 30 min prior to exercise testing. During each exercise trial, participants completed 2 repetitions of bench press at 75% of one-repetition maximum (1RM) as explosively as possible. A linear position transducer was used to measure mean concentric velocity. After 5 min of rest, participants completed 3 sets × repetitions to failure (RTF) at 75% 1RM separated by 2 min of rest between each set. A capillary blood sample was obtained pre- (PRE) and immediately post- (POST) exercise to measure blood concentrations lactate (LA), epinephrine (EPI), and norepinephrine (NE). Mean concentric velocity was significantly higher with GRE when compared to PL (*p* = 0.046). However, total RTF were significantly lower with GRE versus PL (*p* < 0.001). Regardless of treatment, LA was significantly higher Post versus Pre (*p* < 0.001), but GRE resulted in greater Post values compared to PL (*p* = 0.049). EPI and NE increased in both conditions Pre to Post (*p* < 0.001). However, Pre NE was significantly higher with GRE versus PL (*p* = 0.008). Findings indicate that short-term GRE supplementation increases mean bench press velocity but decreases bench press repetition volume. Furthermore, GRE resulted in higher NE levels and blood lactate following exercise. Thus, supplementing with GRE may enhance explosive resistance training performance but may also impair upper body strength-endurance.

## 1. Introduction

*Rhodiola rosea* (Golden Root Extract; GRE) is a high-altitude plant which is native to Europe and parts of Asia [1]. In traditional folk medicine, primary GRE consumption was through chewing or making tea from leaves of the plant in efforts to combat altitude sickness, fatigue, and mood disturbances [2]. Phenolic glycosides contained in GRE, namely salidroside and rosavin, have been identified as the primary active constituents which exert adaptive organismal responses classifying it as an “adaptogen” [1]. Nutritional enrichment with GRE has been linked to hormonal modulation, increased activity of the central nervous system (CNS), and improvements in cellular energy production [3,4]. These physiological responses have been shown to aid in attenuation of various types of stress including mental, metabolic, oxidative, and physical/exercise induced stress [4,5,6]. However, many of the purposed benefits of GRE are unsubstantiated or findings are equivocal especially regarding physical exercise.

While previous evidence has suggested GRE supplementation may have ergogenic benefits, results are mixed leaving the precise efficacy unclear [7]. Acute doses of GRE have been shown to increase time to exhaustion and peak oxygen consumption in physically active males [7]. However, ergogenic effects were not observed with 4-weeks of chronic supplementation. Supporting benefits of acute GRE dosing regimens, Noreen et al. showed that a single dose of GRE improved time trial performance during cycling exercise [8]. Underlying mechanisms for improvements in humans are not well known, but evidence in animal models have suggested improvements in mitochondrial metabolism and increased neural stimulation [4,6]. Abidov et al. reported increases in exercise capacity and mitochondrial ATP levels with GRE supplementation in rodents [6]. Other reports in mice showed increased locomotor activity and CNS excitation with a single dose of GRE supporting possible stimulative properties [4]. Still others have shown little to no benefit of GRE consumption towards exercise performance in humans [9,10]. Jowko et al. showed that a 4-week GRE regimen did not improve endurance exercise performance or physical capacity albeit improvements in reaction time were observed [9]. Likewise, Parisi et al. reported no improvements in perceived exertion or maximal oxygen consumption after chronic GRE supplementation [10]. Disparities in findings may be due to the lack of knowledge related to optimal GRE dosing strategies and exercise training contexts necessitating further study.

Despite limited evidence in the context of anaerobic exercise, GRE is commonly sold and marketed in commercially available supplements for anaerobic and resistance exercise performance. Ryan et al. showed little ergogenic benefit of a multi-ingredient herbal supplement containing GRE, where total work and muscular fatigue were unaffected during isokinetic testing with supplementation [11]. Lowery et al. reported that an 8-week supplementation regimen with a multi-ingredient performance supplement containing GRE increased strength and lean mass in trained males [12]. However, the conjunction of other ingredients with GRE in previous studies leaves it particularly difficult to isolate the exact contribution of GRE to any possible adaptive response. Our lab recently showed that short-term GRE supplementation improved anaerobic sprint performance in trained females [5]. More specifically, anaerobic capacity and power were higher during repeated sprints with GRE treatment compared to a placebo. However, it is unknown if GRE supplementation influences resistance exercise performance and what physiological mechanisms may underpin possible ergogenic effects. The purpose of this study was to examine the impact of short-term GRE supplementation on blood lactate, epinephrine, norepinephrine, and performance during repeated bench press exercise. 

## 2. Materials and Methods

### 2.1. Participants

To determine appropriate sample size, an a priori power analysis was conducted using statistical software (G*power V 3.1.9.4). A previous investigation from our lab measuring anaerobic sprint performance with GRE supplementation showed increases in anaerobic power with an estimated effect size of *d* = 1.07 [13]. To calculate minimal sample size to detect changes in barbell velocity, the following parameters were used: test = *t*-test (matched pairs), d = 1.07, α = 0.05, 1 − *β* = 0.8. This calculated to a minimum sample size range of *n* = 9 for adequate power. Accordingly, a convenience sample of healthy resistance-trained males (*n* = 10) were recruited from the local area. Descriptive characteristics can be seen in (Table 1). Resistance-trained was defined as engaging in resistance exercise, bench press in particular, ≥2–3 days each week [14]. Screening for suitability of exercise was determined using a physical activity readiness questionnaire (PARQ) [14]. Exclusion criteria included: upper body injuries within the past six months, a current disease or diagnosis which limited exercise capacity, and current supplementation with GRE or any of its constituents [5]. Before each visit, participants were asked to refrain from vigorous activity 24 h prior and from consuming caffeine, nicotine, and alcohol 12 h prior [5]. Participants were asked to maintain their normal sleep and dietary routines prior to each visit. Compliance was self-reported. 

### 2.2. Study Design

**Figure 1:**
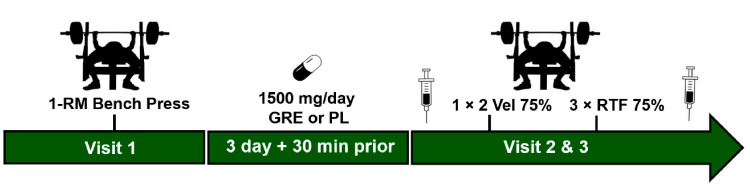
Study design. For the first visit, a one-repetition maximum (1-RM) for bench press was obtained for each participant. In a crossover counterbalanced manner, participants supplemented with GRE or Placebo (PL) for 3-days and took an additional dose 30 min prior to each visit. Blood (syringes) was collected before exercise (Pre) and after exercise (Post). For each visit, participants completed 1 set × 2 explosive reps at 75% of 1-RM to measure velocity (Vel). Following this, participants completed 3 sets × Repetitions to failure (RTF).

The study design is shown in (Figure 1). The current investigation used a double-blinded, counterbalanced, crossover study design to examine the effects of short-term GRE supplementation on resistance exercise performance and associated responses of blood lactate (LA), epinephrine (EPI), and norepinephrine (NE). Resistance-trained males volunteered to participate and completed two bench press trials each with a different randomized condition: (1) GRE, (2) placebo (PL; gluten-free cornstarch). Following supplementation, participants completed a series of bench press sets while measurements were collected for mean barbell velocity and total volume performed. Blood was collected immediately prior to (Pre) and immediately after (Post) exercise to measure LA, EPI, and NE. Each trial was separated by a 1-week washout period.

### 2.3. Supplementation

For the intervention, participants were supplemented with either PL (gluten-free cornstarch) or GRE as previously described by our group [5]. Briefly, a commercially available GRE contained a standardized extract to a minimum of 3% total Rosavins and 1% total Salidroside (NOW Food Inc., Bloomingdale, IL, USA). Leading up to each trial, participants ingested 500 mg of corresponding treatment three times daily (~1500 mg/day) for a total of three days prior to the exercise trial [5]. An additional 500 mg dose was taken thirty minutes prior to each trial. All treatments were distributed in a double-blinded manner whereby an independent researcher organized non-identifiable opaque bags containing each treatment. To ensure compliance, empty bags were returned by participants and recorded. Additionally, all participants were asked to replicate their diet each day of the exercise trials. Participants were not aware of any experimental hypotheses and no side effects from supplementation were reported.

### 2.4. Blood Collection and Analysis (Lactate, Epinephrine, Norepinephrine)

Approximately 500–600 μL of capillary blood was collected Pre and Post exercise through a finger prick. A 17-gauge 2.0 mm depth disposable lancet was used to induce bleeding on either the third or fourth finger. The first drops of blood were used to measure blood lactate using a lactate meter (Lactate Plus Meter, Nova Biomedical, Waltham, MA, USA). Then, a massage technique was used to promote bleeding and the remaining volume of blood was collected via capillary action into lithium-heparin coated microvette^®^ tubes (SARSTEDT, Newton, NC, USA). Whole blood was then centrifuged at 10,000 rpm for 10 min, plasma was decanted, and subsequently stored at −80° C until biochemical analysis which was completed following the conclusion data acquisition. Plasma concentrations of EPI and NE were determined using a commercially available enzyme-linked immunosorbent assay (ELISA) kit (ABNOVA, Taipei, Taiwan) [15,16]. All samples were analyzed in duplicate and according to the manufacturer’s instructions. 

### 2.5. One Repetition Maxium (1-RM) and Familiarization

For the first visit, maximal upper body strength was measured via one-repetition maximum (1-RM) testing [17,18]. Participants completed a low intensity bench press warm-up according to American College of Sports Medicine (ACSM) recommendations [14]. Following the warm-up, the barbell weight was progressively increased by 2.5–20.0 kg for one attempt until the participant could not complete the concentric phase of the bench press. The 1-RM load was determined within four attempts with a rest period of three to five minutes between attempts [19,20]. Following this, familiarization with the protocol of lifting as explosively as possible was completed. Participants were asked to lift a 20-kg Olympic barbell as quickly and explosively as possible for three repetitions which was repeated for a total of three sets. During repetitions, participants were instructed to bring the bar to their chest and end at full extension. Form was corrected if needed.

### 2.6. Procedures

For each exercise trial, a Pre exercise capillary blood sample was obtained as previously described. Following this, participants completed a warm-up consisting of 5 repetitions at 40% of 1RM and 3 repetitions at 60% of 1RM with each set separated by a 2-min rest period [18]. Participants then completed 1 set × 2 repetitions of bench press at 75% of 1-RM with maximum explosive intent. During this set, a linear position transducer (GymAware, Kinetitech Performance Technology, Australia) was attached to the barbell to measure mean concentric velocity. This device has been previously validated for velocity measurements by multiple groups [21,22]. In addition, our group has previously used this equipment with excellent test–retest reliability in our laboratory (ICC = 0.932) [19,23]. The device was used according to manufacturer’s instructions such that the device was attached to the barbell with a perpendicular angle being achieved throughout the lift [24]. Mean barbell velocity was averaged across the 2 repetitions and used for analysis. Following a 5-min rest period, participants then completed 3 sets × repetitions to failure (RTF) of bench press exercise at 75% 1-RM. Each set of RTF was separated by 2 min of rest. Failure was determined by the participants’ incapacity to finish the concentric phase of the lift. Repetitions for each set and total repetitions were recorded for analysis. Immediately Post exercise, blood collection protocols were repeated.

### 2.7. Data Analysis

Data analysis was completed using Jamovi software (Version 0.9; Sydney, Australia). Confirmation of data normality was conducted using the Shapiro–Wilk methods. Mean barbell velocity and total RTFs were analyzed using a paired samples *t*-test. Set-to-set RTFs was analyzed using a 2 × 3 [Treatment × Set] repeated measures ANOVA. Blood LA, EPI, and NE were analyzed using a 2 × 2 [Treatment × Time] repeated measures ANOVA. Bonferroni-Holm post hoc analysis was conducted as warranted. For significant main effects, individual means post hoc analysis was performed as previously recommended by Wei et al. [25]. Estimates of effect size for main effects were calculated using eta squared (η^2^) and interpreted as: 0.01—small; 0.06—medium; ≥0.14—large [26,27]. Individual mean effect sizes were calculated via Cohen’s d (d) between conditions and interpreted as: 0.2—small; 0.5—moderate; 0.8—large [26,27]. Significance was set at *p* ≤ 0.05 a priori.

## 3. Results

### 3.1. Mean Velocity and Repetitions to Failure 

**Figure 2:**
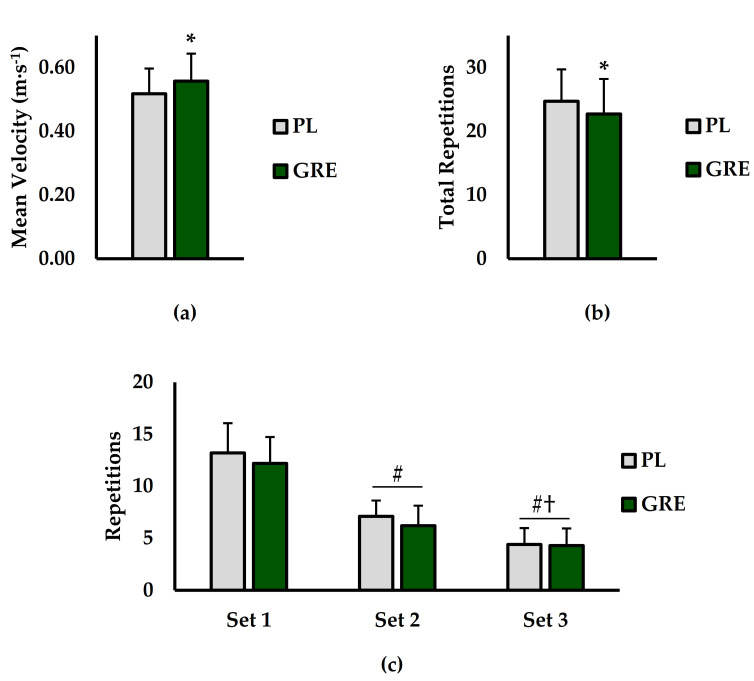
(**a**) Mean velocity (m·s^−1^), (**b**) Total repetitions to failure (total repetitions), and (**c**) Set-to-set repetitions to failure (repetitions) compared between placebo (PL; grey bars) and golden root extract (GRE; green bars). * indicates significantly different from GRE (*p* < 0.05). # indicates significantly different from set 1 (*p* < 0.05). † indicates significantly different from set 2 (*p* < 0.05).

Mean velocity, total RTF, and set-to-set RTF analysis is shown in (Figure 2). For mean velocity (m·s^−1^; Figure 2a), GRE supplementation resulted in significantly higher mean velocity compared to PL (*p* = 0.049; *d* = 0.728). However, total RTF (reps; Figure 2b) were significantly lower in the GRE condition versus PL (*p* < 0.001; *d* = 1.90). For set-to-set RTF (reps; Figure 2c), there was a significant main effect for treatment (*p* < 0.001; η^2^ = 0.007) and set (*p* < 0.001; η^2^ = 0.755) but no interaction for treatment × set (*p* = 0.195; η^2^ = 0.002). Post hoc analysis for treatment revealed that RTFs were lower for GRE versus PL (*p* < 0.001). For set, significantly more repetitions were completed during set 1 versus set 2 (*p* < 0.001) and set 3 (*p* < 0.001). Furthermore, RTF were significantly higher for set 2 versus set 3 (*p* < 0.001). 

### 3.2. Blood Lactate, Epinephrine, Norepinephrine

**Figure 3:**
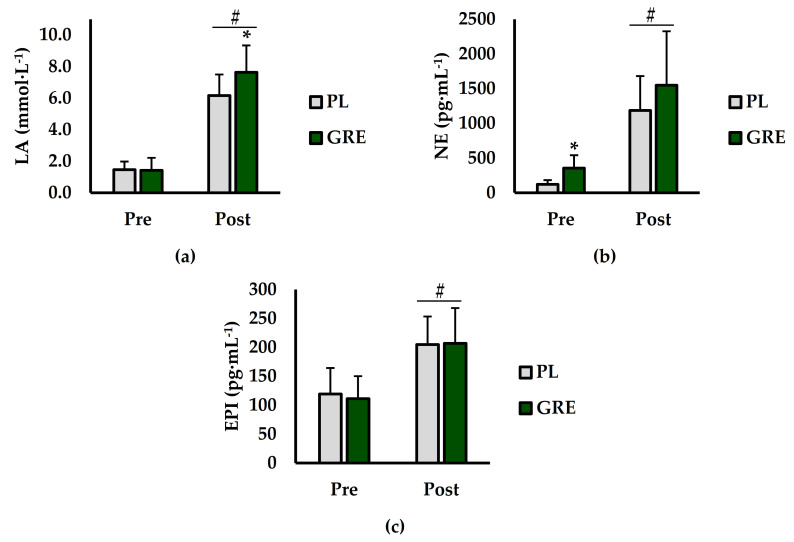
(**a**) Blood lactate (LA; mmol·L^−1^), (**b**) Plasma Norepinephrine (NE; pg·mL^−1^), and (**c**) Plasma Epinephrine (EPI; pg·mL^−1^), compared between placebo (PL; grey bars) and golden root extract (GRE; green bars). Additionally, measurements were taken and compared immediately prior to (Pre) and after (Post) exercise. * indicates significantly different from GRE (*p* < 0.05). # indicates significantly different from Pre (*p* < 0.05).

Analysis of concentrations of blood LA (mmol·L^−1^), norepinephrine (pg·mL^−1^), and epinephrine (pg·mL^−1^) are shown in (Figure 3). For blood LA (Figure 3a), there was a main effect for treatment (*p* = 0.047; η^2^ = 0.017) and time (*p* < 0.001; η^2^ = 0.830). A significant interaction for treatment × time (*p* = 0.041; η^2^ = 0.019) was also observed. Specifically, blood LA was higher Post compared to Pre (*p* < 0.001) and higher with GRE supplementation compared to PL (*p* = 0.047). At the Post time point, blood lactate was significantly higher for GRE versus PL (*p* = 0.049). For NE (Figure 3b), there was a main effect for treatment (*p* = 0.018; η^2^ = 0.047) and time (*p* < 0.001; η^2^ = 0.588) but no interaction for treatment × time (*p* = 0.659; η^2^ = 0.002). Specifically, GRE supplementation resulted in higher NE levels than PL (*p* = 0.018) and NE levels were higher at Post compared Pre (*p* < 0.001). For the Pre time point, NE levels were significantly higher with GRE than PL (*p* = 0.008). Analysis of EPI (Figure 3c) showed a significant main effect for time (*p* < 0.001; η^2^ = 0.485) but not for treatment (*p* = 0.882; η^2^ = 0.001). Furthermore, there was no interaction for treatment × time (*p* = 0.727; η^2^ = 0.002). EPI was significantly higher at Post compared to Pre (*p* < 0.001).

## 4. Discussion

Previous evidence on GRE supplementation and exercise has been primarily completed in endurance-based activities and has revealed mixed results [7,9,10]. Other studies have suggested strength adaptations may be potentiated with multi-ingredient supplements containing GRE but contributions towards performance are unclear due to the amalgam of constituents [12]. Our lab recently showed short-term GRE supplementation improved anaerobic capacity and power during repeated sprints [5]. However, it is currently unknown if GRE supplementation influences resistance exercise performance and what physiological mechanisms may facilitate any enhancements. Current findings show that short-term GRE enrichment increases mean barbell velocity during bench press exercise. However, total repetition volume suffered compared to PL. While blood LA increased in both GRE and PL groups following exercise, LA was higher after exercise with GRE treatment. Both Epi and NE increased following exercise regardless of treatment, but NE was significantly higher with GRE supplementation especially prior to exercise. These results suggest short-term GRE supplementation increases explosive resistance exercise performance and may have sympathomimetic properties. However, GRE decreased strength-endurance and increased post-exercise blood LA suggesting a possible exacerbation of fatigue.

Present data show that short-term GRE supplementation resulted in a significant ~8% increase in mean barbell velocity versus PL. This is bolstered by past evidence from our lab showing improved anaerobic power during repeated Wingate anaerobic sprint tests with an identical GRE dosing regimen [5]. Still, others have shown little to no effects with GRE supplementation. Disagreements between findings may be in part due to differences in supplementation protocols. Multiple investigations have shown that continuous long-term GRE supplementation over periods of weeks may not have any ergogenic advantage compared to PL [7,10,28]. As such, it appears that acute or short-term period of GRE enrichment are most efficacious [5,7,8]. For example, De Bock et al. reported that a single acute dose of GRE resulted in increased peak oxygen consumption and endurance exercise capacity while 4-weeks of supplementation showed no apparent benefit [7]. While speculative, long-term supplementation may induce desensitization and tolerance to GRE as is common with many other purported stimulants [29]. Future longitudinal investigations with serial measurements on how GRE influences exercise performance are needed to determine optimal dosing strategies. While mechanisms for increases in explosive performance currently observed remain to be fully elucidated, performance increases in this capacity may be due to increased CNS and/or sympathetic activity. Indeed, Perfumi et al. reported increased exercise capacity and CNS activity in rodents treated with GRE [4]. Supporting this in humans, GRE has been shown to improve simple and choice reaction time which may reflect CNS modulation [9]. Whether present improvements in barbell velocity can indeed be attributed to CNS stimulation is unclear, but previous work from our lab and others have shown that CNS stimulants significantly increase barbell velocity and power during upper and lower body resistance exercises [13,23]. GRE may also have sympathomimetic qualities as partially evidenced by current increases in NE compared PL. Of particular importance, NE levels were ~34% higher prior to exercise with GRE treatment compared to PL. Catecholamines and adrenergic receptor activation have been shown to alter skeletal muscle force and contraction [30]. Anticipatory catecholamine responses have been linked to improved muscular performance [31]. Adrenergic receptor stimulation has been well described to cause increases in muscular force in both fast and slow fiber types [30,32]. This is likely mediated through increased cytosolic calcium release thereby allowing for greater actin-myosin interaction [30]. Thus, it is plausible that higher NE levels currently observed prior to bench press velocity measurements resulted in adrenergic-mediated increases in cross-bridge formation allowing for greater muscular force and velocity of movement. Furthermore, sympathetic stimulation has been linked to faster relaxation rate of slow-twitch muscle fibers [33]. This could in turn allow for faster initiation of subsequent muscular contractions during movement thereby altering movement velocity. However, the reader is cautioned that these mechanisms cannot be fully supported by current data alone and more mechanistic studies are necessary to investigate how GRE might influence muscular contraction.

Interestingly, total repetition volume was significantly diminished by ~8% with GRE treatment compared to PL although set-to-set differences did not exist. While many investigations have suggested little to no performance improvement with GRE supplementation, this appears to be the first to show performance decrement and reasons for this are not fully evident. This may be due to noted increases in catecholamines presently albeit adrenergic influences on skeletal muscle and fatigue are not fully known. Frye et al. showed in vitro that skeletal muscle fibers bathed in solutions containing NE resulted in lower rate of force development independent of blood flow [34]. It was also noted that similar α_1_-agonists initiated similar acceleration in fatigue. This may in part be due to alterations in glycolytic metabolism. Blockade of α and β adrenergic receptors have been linked to decreased glycolytic flux and Na^+^/K^+^ ATPase pump function [35]. Furthermore, intravenous NE infusion has been shown to increase lactate production from skeletal muscle [36]. Current data may support these findings as blood lactate and NE were concomitantly increased with GRE supplementation. It is plausible that observed LA increases with GRE reflect accelerated glycolysis and hydrogen ion production ultimately leading to increased fatigue. Accumulation of hydrogen ions has been suggested as a primary mechanism of fatigue during resistance exercise [37]. Thus, decreases in repetition volume observed presently may be due to the aforementioned mechanisms although this will need to be empirically confirmed. 

While the present study shows novel information regarding ergogenic potential of GRE supplementation, there were several limitations. First, a relatively small and homogenous sample was used currently. It is not known if these results will fully translate to untrained, different aged, or female counterparts. However, previous work from our lab using an identical GRE regimen showed increased anaerobic performance in females [5]. Additionally, it is still unknown as to what is the optimal dosing GRE regimen for athletic performance. While current findings support GRE use for explosive performance, the opposite is true for muscular strength-endurance. In parallel to this, it is uncertain as to how chronic or periodic GRE supplementation regimens may influence long-term strength adaptations and hypertrophy. Much more research is needed to strategically identify the most appropriate regimens for various types of exercise and intensities.

## 5. Conclusions

In conclusion, short-term GRE supplementation increased barbell velocity but resulted in decreased total repetition volume during bench press exercise. GRE supplementation also resulted in higher blood LA and NE levels while EPI was unchanged by treatment. From a practical standpoint, bench press is commonly used in strength and conditioning programs to increase upper body muscular strength and power. Current data suggest individuals looking to acutely improve explosive ability may benefit from GRE treatment. However, GRE may not be beneficial for repeated exercise or those looking to maximize repetition volume and caution should be used when supplementing with GRE to improve strength-endurance.

## Figures and Tables

**Table 1:** Descriptive characteristics of participants (*n* = 10).

Characteristic	Mean ± SD
Age (years)	24.8 ± 5.6
Height (cm)	178.6 ± 7.1
BM (kg)	83.2 ± 7.6
Training Experience (years)	8.7 ± 6.3
1-RM (kg)	114.2 ± 15.8
Relative BP Strength [1-RM (kg)/BM (kg)]	1.4 ± 0.2

BM (Body mass); 1-RM (1-Repetition Maximum); BP (Bench Press).

## Data Availability

All data are available within this manuscript.
